# Dormancy-Associated MADS-Box (*DAM*) Genes Influence Chilling Requirement of Sweet Cherries and Co-Regulate Flower Development with *SOC1* Gene

**DOI:** 10.3390/ijms21030921

**Published:** 2020-01-30

**Authors:** Jiyuan Wang, Zhen Gao, Hui Li, Songtao Jiu, Yueting Qu, Lei Wang, Chao Ma, Wenping Xu, Shiping Wang, Caixi Zhang

**Affiliations:** Department of Plant Sciences, School of Agriculture and Biology, Shanghai Jiao Tong University, Minhang, Shanghai 200240, China; 016150910029@sjtu.edu.cn (J.W.); gaoz89@126.com (Z.G.); oldhorseli@sjtu.edu.cn (H.L.); jiusongtao@sjtu.edu.cn (S.J.); lahmmuller@hotmail.com (Y.Q.); leiwang2016@sjtu.edu.cn (L.W.); chaoma2015@sjtu.edu.cn (C.M.); wp-xu@sjtu.edu.cn (W.X.); fruit@sjtu.edu.cn (S.W.)

**Keywords:** sweet cherry, dormancy, chill requirement, warm winter, flower development

## Abstract

Floral bud dormancy release of fruit tree species is greatly influenced by climate change. The lack of chilling accumulation often results in the occurrence of abnormal flower and low yields of sweet cherries (*Prunus avium* L.) in warm regions. To investigate the regulation of dormancy in sweet cherries, six *DAM* genes with homology to peach *DAM*, designated *PavDAM1-6*, have been identified and characterized. Phylogenetic analysis indicate that these genes are similar to *DAMs* in peach, apple and pear. The expression patterns of the *PavDAMs* in the low-chill cultivar ‘Royal Lee’ were different from that in the high-chill cultivar ‘Hongdeng’. ‘Royal Lee’ exhibits lower transcriptional level of *PavDAM1* compared to ‘Hongdeng’, especially at the stage of chilling accumulation, and transcriptional levels of *PavDAM4/5* were high in both cultivars during the endodormancy. Ectopic expression of *PavDAM1* and *PavDAM5* in *Arabidopsis* resulted in plants with abnormal flower and seed development, especially the *PavDAM5*. Higher transcriptional levels of *SOC1* were observed in transgenic *PavDAM1/5* lines, and ectopic expression of *PavSOC1* had the similar floral phenotype. Further, protein interaction analysis demonstrated that PavDAM1/5 could interact with PavSOC1 in vivo and in vitro, which will help clarify the molecular mechanism of the flower development in sweet cherry or other fruit trees.

## 1. Introduction

Floral bud dormancy is an important biological process allowing sweet cherries (*Prunus avium* L.) to survive in winter. The dormancy has three main phases: paradormancy, endodormancy and ecodormancy [1]. Endodormancy plays a pivotal role for the chilling accumulation in the dormant phases. After adequate chill, endodormancy is released and enters into the ecodormancy [2]. If the conditions are favourable in the ecodormancy, the floral buds will break following the spring [3]. However, temperate fruit tree species that do not require sufficient chilling show low budburst and delay of the flowering date in warm climates [4,5]. Our previous report showed that the high-chill sweet cherry cv. Hongdeng, have a high percentage of abnormal female floral organs in warm regions because of a lack of chilling accumulation during the endodormancy, but not in cool regions [6]. Recently, we found that expression of MADS-Box genes were also associated with the formation of polycarpy and fruit doubling under high temperature in sweet cherries [7].

DORMANCY-ASSOCIATED MADS-BOX (*DAM*) genes have been investigated widely in many perennial fruit species, such as peach, apple, pear and apricot [8,9,10,11,12]. Six tandemly arrayed MADS-box genes are found in peach, belonging to the *SVP/AGL24* clade. They are called *PpDAM1-6*, relating to the dormancy breaking [8,13]. Most of the genes are highly expressed in winter, and down-regulated towards spring in peach; similar expression patterns are also found in other fruit species. *DAM5* and *DAM6* transcript levels are different between the high- and low-chill cultivars, suggesting a disparate function during dormancy release in peach [14,15]. Transgenic studies demonstrate that some of the *DAMs* induce growth cessation and bud formation in the apple and Japanese apricot [16,17,18]. In Chinese cherry (*Prunus pseudocerasus*), *PavDAM4-6* have been also isolated and analysed by the RNA-Seq in dormant floral buds [19]. However, few studies in molecular level to clarify the relationship between chilling requirement and dormancy have been reported in sweet cherries in warm regions.

The interaction between *DAMs* and other genes are associated with the growth inhibition and bud set during the dormancy. The cold response genes C-repeat binding factors (*CBFs*) regulate the *DAMs* expression levels by binding to the DRE/CRT (dehydration-responsive element/C-repeat) cis-acting element in *DAM* promoters. The *DAMs* inhibit *FT2* (*FLOWERING LOCUS T*) expression to induce endodormancy in pear [11,20]. Moreover, *PmDAM6* has been identified to interact with *PmSOC1* (*SUPPRESSOR OF OVEREXPRESSION OF CO1*), suggesting that *PmSOC1* participate in changes of dormancy status and blooming date in Japanese apricot [21]. In *Arabidopsis*, the function of *AtSOC1* is associated with the development of floral organs and early flowering as an integrator in the floral transition [22]. *At*S*OC1* interacts with *AtAGL24* (*AGAMOUS-LIKE 24*), a homolog of *DAM* [23,24]. In contrast to *SOC1* in annual herbaceous plant *Arabidopsis*, *SOC1* represses flowering and affects the duration of dormancy in perennials herbaceous strawberry [25]. In addition, the protein–protein interactions are verified among PmDAM1, PmDAM6 and PmDAM5 proteins in the *Prunus mume* [12]. However, the interaction of *DAMs* and *SOC1* in floral buds dormancy and development of fruit species, including sweet cherries, has not been reported. To understand the mechanism of *DAM*-mediated regulation of sweet cherry bud dormancy and development, we investigated the relationship between chilling accumulation and bud dormancy release, and the interaction of *DAMs* and *SOC1* in floral buds development of sweet cherries. 

## 2. Results

### 2.1. Difference of Dormancy Status and Chilling Requirement between ‘Royal Lee’ and ‘Hongdeng’

**Figure 1:**
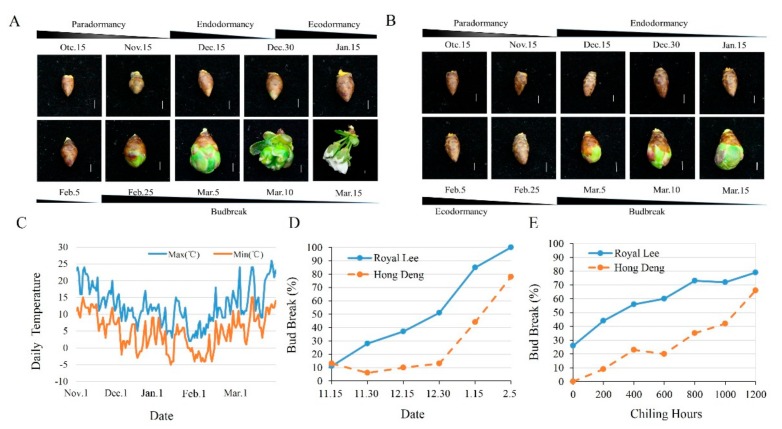
Dormancy status and chilling hours of floral buds of ‘Royal Lee’ and ‘Hongdeng’. (**A**) The dormancy status of the ‘Royal Lee’ in the experimental farm. Bars, 2.5 mm. (**B**) The dormancy status of the ‘Hongdeng’ in the experimental farm. Bars, 2.5 mm. (**C**) The temperature from Nov. 1 to Mar. 30 in the experimental farm. (**D**) Bud break percentage of ‘Royal Lee’ and ‘Hongdeng’ collected in the experimental farm after 21 d of forcing conditions. (**E**) The chilling requirements for bud break of the two cultivars collected from the 4 °C storage house every 200 h.

The dormancy status and chilling requirement for bud break are cultivar-dependent in sweet cherries. As shown in Figure 1A,B, the progress of dormancy release in ‘Royal Lee’ is faster than that of ‘Hongdeng’. The length of endodormancy in ‘Royal Lee’ and ‘Hongdeng’ was about 30 (1–30, December) and 60 (1 December–23 January) days, respectively. Chill accumulation for both cultivars started from 18 November according to the 0–7.2 °C model [26]. Then the temperatures declined and maintained a low level until the early February (Figure 1C). The percentage of floral bud burst in ‘Hongdeng’ still remain at 10% until 30 December, however, it reached 50% in ‘Royal Lee’, which indicated the end of endodormancy. In ‘Hongdeng’, it sharply increased after 30 December and reached 50% until 23 January (Figure 1D). The evaluation of chilling requirement showed that ‘Royal Lee’ had about 400 chilling hours (CH), while ‘Hongdeng’ had about 1200 CH (Figure 1E). Obviously, the chilling requirement of ‘Royal Lee’ was much lower than that of ‘Hongdeng’. Our results confirmed that ‘Royal Lee’ is a low-chill cultivar, and ‘Hongdeng’ is a high-chill cultivar.

### 2.2. Identification and Phylogenetic Analysis of Six PavDAM Genes in Low- and High-Chill Cultivars

**Figure 2:**
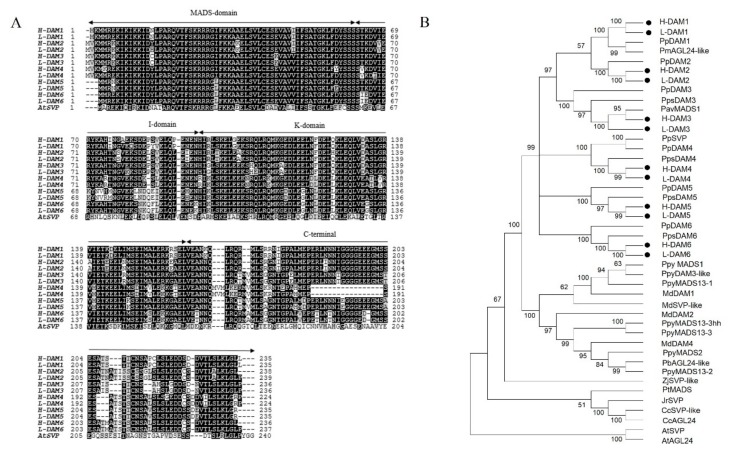
Six *PavDAM* genes in sweet cherry. (**A**) Alignment of the sequences of *PavDAMs* of ‘Hongdeng’ and ‘Royal Lee’, and *SVP* of Arabidopsis. MADS box, K box, I region domains and C terminal are indicated by arrows. ’H-DAMs’, ‘Hongdeng’; ’L-DAMs’, ‘Royal Lee’. (**B**) Phylogenetic tree based on the amino acid alignment of the two cultivars DAM proteins marked with dots and other plant species. The number at each branch indicates the bootstrap value of 1000 replicates, and branches with more than 50% bootstrap values are shown. At, *Arabidopsis thaliana*; Cc, *Carya cathayensis*; Jr, *Juglans regia*; Md, *Malus x domestica*; Pav, *Prunus avium*; Pb, *Pyrus x bretschneideri*; Pm, *Prunus mume*; Pp, *Prunus persica*; Pps, *Prunus pseudocerasus*; Ppy, *Pyrus pyrifolia*; Pt, *Populus tomentosar*; Zj, *Ziziphus jujube*.

To isolate the full-length cDNAs of the six *PavDAM* genes, 1 μg of total RNA of two sweet cherry cultivars was respectively converted into cDNA and were subsequently diluted five times with sterile water. Primers were designed using Primer 5 software according to *DAM* homologs of peach and Chinese cherry [8,19], and sweet cherry reference genome (http://cherry.kazusa.or.jp/). Six dormancy-associated MADS-box (DAM) genes in sweet cherries could be called *PavDAM1* to *PavDAM6*. The six PavDAMs proteins have high homology and almost no distinct difference between ‘Hongdeng’ and ‘Royal Lee’. They have similar genomic structures which are made up of the MADS box domain at the N-terminal end, I box domain, K box domain at the middle position, and C-terminal, which are similar to *Arabidopsis SVP* and *AGL24*, revealing that *PavDAMs* are MIKC^c^-type MADS box genes (Figure 2A).

Phylogenetic analysis showed that these six genes *PavDAMs* in ‘Hongdeng’ and ‘Royal Lee’ were closely related to each other, and formed orthologous pairs with Chinese cherry and peach DAM proteins (Figure 2B). GenBank accession numbers had been included in Table S2. *PavDAMs* were most closely related to *SVP/AGL24*, and belonged to the *SVP/AGL24* clade of angiosperm MADS box genes. PavDAM1 are likely orthologues of PmAGL24-like protein, similarly, PavDAM4 are likely orthologues of PpSVP. PavDAMs were also closely related to the SVP proteins of woody perennial species from a separate sub-clade, jujube and walnut, along with independent sub-clade *Arabidopsis* (Figure 2B).

### 2.3. Expression Analysis of Six PavDAM Genes in Low- and High-Chill Cultivars 

**Figure 3:**
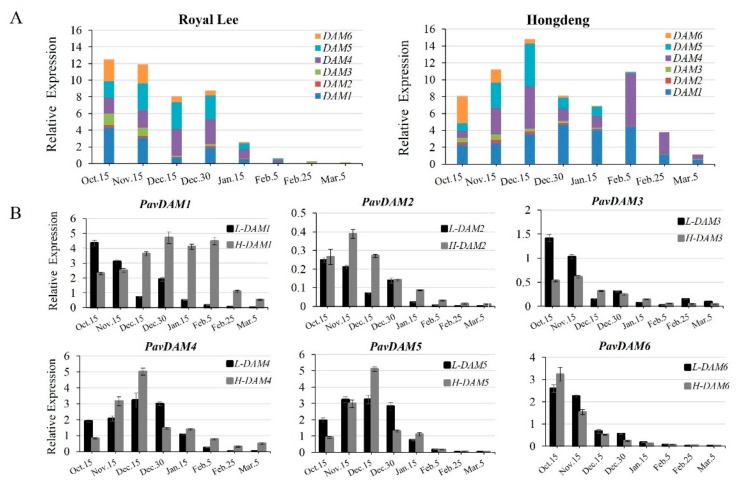
Expression profiling of *PavDAMs* in the floral buds of two sweet cherry cultivars. (**A**) The expression patterns of six *PavDAMs* in ‘Royal Lee’ and ‘Hongdeng’ respectively. ’H-DAMs’, ‘Hongdeng’; ’L-DAMs’, ‘Royal Lee’. (**B**) Comparison of the expression patterns between each pair of *PavDAMs* in ‘Royal Lee’ and ‘Hongdeng’. Error bars represent SE of three biological replicates.

To investigate the differences in the expression profile between the low- and high-chill cultivar, we detected seasonal expression changes of *PavDAMs* in the floral buds from both cultivars by real-time RT-PCR analysis. The expression of the *PavDAMs* maintained high levels from 15 October to 30 December in the low-chill cultivar ‘Royal Lee’, while it is from 15 October to 5 February in ‘Hongdeng’. It indicated that compared with the earlier budbreak in ‘Royal Lee’, the delayed budbreak of ‘Hongdeng’ coincides with a longer duration of high transcript levels of *PavDAMs* in winter (Figure 3A).

The transcript levels of *PavDAM2, PavDAM3* and *PavDAM6* were lower in both cultivars during the winter period, compared with the high transcript levels of *PavDAM1*, *PavDAM4* and *PavDAM5* (Figure 3A). The expression pattern of the *PavDAM4/5* in the low-chill cultivar ‘Royal Lee’ was similar to that in the high-chill cultivar ‘Hongdeng’ (Figure 3B). The transcript levels of the *PavDAM4/5* in both cultivars began to increase and reached the peak on 15 December in early winter. Subsequently, it started to decrease gradually toward the spring. It was worth noting that the expression pattern of *PavDAM1* was different between the two cultivars (Figure 3B). The transcript level of *PavDAM1* was high in the winter, but its expression level in the low-chill cultivar rapidly decreased compared with the high-chill cultivar. However, they still remained at a high transcript levels in high-chill cultivar during the later stage of dormancy (Figure 3B).

### 2.4. Subcellular Localization of Six PavDAMs

**Figure 4:**
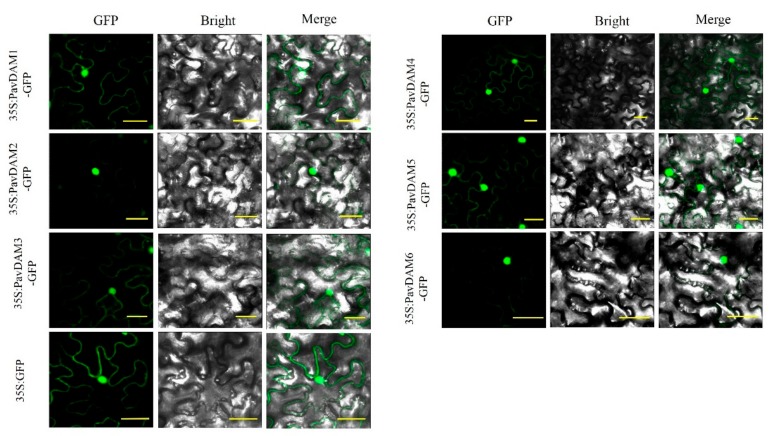
Subcellular localization of six *PavDAMs* from ‘Hong deng’. Leaves of *Nicotiana benthamiana* plants expressing 35S:PavDAMs-GFP and 35S:GFP. Scale bar = 30 μm.

To examine the function of *PavDAMs*, their subcellular localization were measured by the fluorescent protein-tagging method first. Our results showed that while green fluorescent protein (GFP) alone presented a dispersed cytoplasmic distribution, GFP-tagged PavDAMs were located in the nucleus and cytomembrane (Figure 4). These results suggest that PavDAMs might be the transcription factors.

### 2.5. Ectopic Overexpression of PavDAM1/5 Affect Flower Development in Arabidopsis

**Figure 5:**
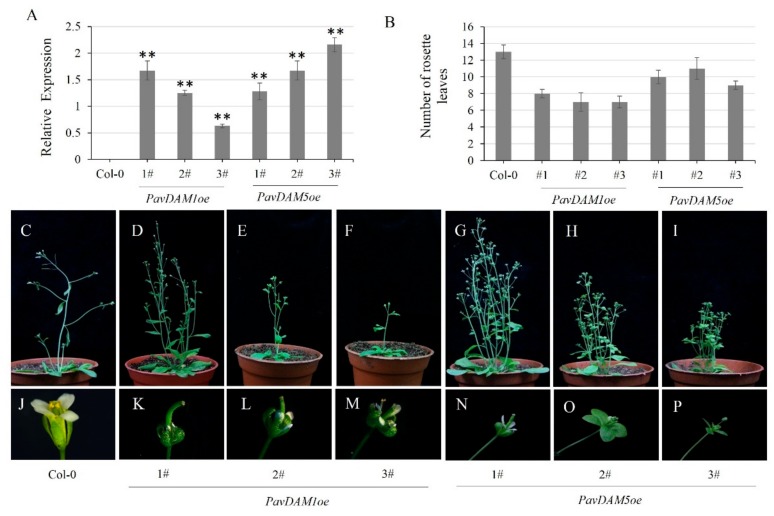
Constitutive expression of *PavDAM1/5* affects flower development in *Arabidopsis*. (**A**) Relative expression of *PavDAM1/5* in transgenic and control plants. (**B**) Number of rosette leaves in transgenic and control plants. (** *p* ≤ 0.01; Student’s *t*-test). (**C**,**J**) Normal flower development of wild-type *Arabidopsis*. (**D**–**I**) Phenotypes of transgenic *Arabidopsis PavDAM1/5* plants. (**K**–**P**) Abnormal flower development in lines expressing.

To identify the function of *PavDAMs* mediated in flower development of sweet cherries, transgenic *Arabidopsis* were generated using the *PavDAM1/5* full-length cDNAs driven by the CaMV 35S promoter. The levels of *PavDAM1/5* transgene expression were confirmed in three independent transgenic lines, compared with wild-type (Col-0) (Figure 5A). The number of rosette leaves in the *35S: PavDAM1/5* transgenic lines was only 7–11, while the Col-0 was 13–14 (Figure 5B). Obvious differences in flower phenotype were observed between the transgenic lines and Col-0 (Figure 5C–P). In transgenic lines, the flowers showed abnormal phenotype, such as, cincinal sepals, flowers with large sepals, wrinkled and short stigmas, and narrow sepaloid petals. Sterile flowers were found in the *PavDAM5oe-3#* lines (Figure 5I, P). However, ectopic overexpression of *PavDAM4* in *Arabidopsis* did not induce the abnormal floral development (Figure S1A–D). And the levels of *PavDAM4* transgene expression were confirmed in three independent transgenic lines, compared with Col-0 (Figure S1E). As a result, *PavDAM1/5* genes have the potential influence on flower development.

### 2.6. Relative Expression of SOC1 in Arabidopsis and Sweet Cherries

**Figure 6:**
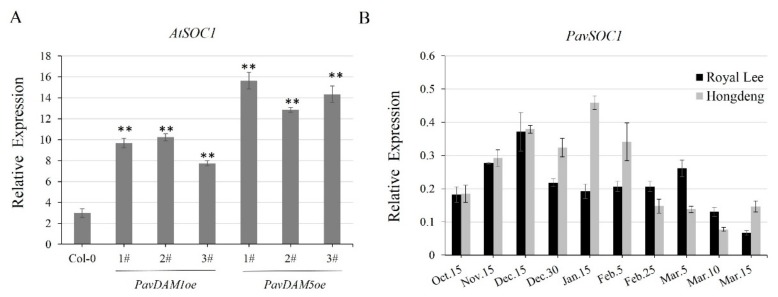
Relative expression of *SOC1* in *Arabidopsis* Col-0 and transgenic lines (*AtSOC1*, **A**) and sweet cherry (*PavSOC1*, **B**), normalized to respective ACTIN. Error bars represent standard errors (SE) for three replicates. (** *p* ≤ 0.01; Student’s *t*-test).

To associate further the molecular mechanism of *PavDAMs* regulating flower development, our previous results in the screening of the genes involved in flowering showed that a higher transcript level of *AtSOC1* was observed in the *PavDAMs* transgenic lines rather than in Col-0 (Figure 6A). Furthermore, *PavSOC1* demonstrated increased expression throughout the winter dormancy period and decreased expression toward the spring in sweet cherries (Figure 6B). Expression patterns of *PavSOC1* in the sweet cherries were similar to those of *PavDAMs* during the stages of dormancy and dormancy release. 

### 2.7. Ectopic Overexpression of PavSOC1 in Arabidopsis

**Figure 7:**
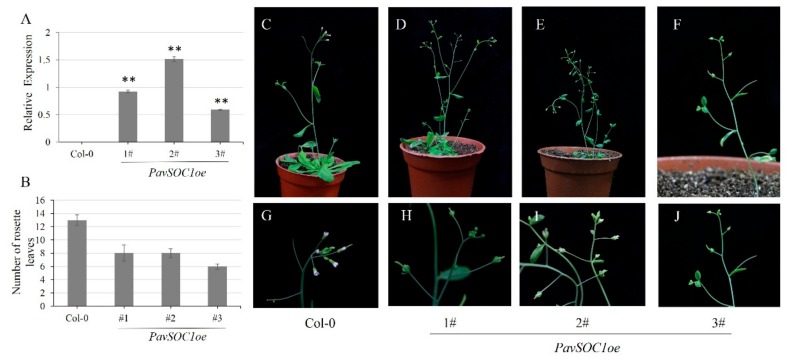
Constitutive expression of *PavSOC1* affects flower development in *Arabidopsis*. (**A**) Relative expression of *PavSOC1* in transgenic and control plants. (**p ≤ 0.01; Student’s *t*-test). (**B**) Number of rosette leaves in transgenic and control plants. (**C**, **G**) Normal flower development of wild-type *Arabidopsis*. (**D-F**, **H-J**) Abnormal flower development in *PavSOC1oe Arabidopsis* plants.

Because of the higher transcript level of *AtSOC1* observed in the *PavDAMs* transgenic lines and similar expression patterns as well as *PavDAMs* in sweet cherries, ectopic overexpression of *PavSOC1* in *Arabidopsis* were performed. We observed similar phenotype with the *PavDAM1oe* and *PavDAM5oe* lines, including the larger and flexuous calyces, compared with the Col-0 (Figure 7C–J). Transgenic lines were further verified by qRT-PCR (Figure 7A). The number of rosette leaves in the *35S: PavSOC1* transgenic lines was only 6–8, while the Col-0 was 13–14 (Figure 7B), indicating an early flowering phenotype in transgenic lines. Therefore, the gene *PavSOC1* has the potential role in regulating the floral development in *Arabidopsis*. 

### 2.8. DAM Proteins Interact with SOC1 Protein In Vitro and In Vivo in Sweet Cherries

**Figure 8:**
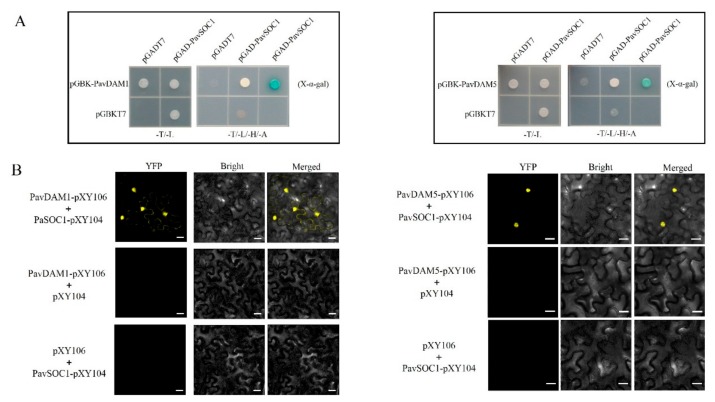
Interactions between PavDAM1/5 and PavSOC1 protein in vitro and in vivo. (**A**) Yeast two-hybrid assay. pGBK-PavDAM1 or pGBK-PavDAM5 interacted with pGAD-PaSOC1 conferred Y2HGold cell growth on SD/-Leu/-Trp/-His/-Ade plates. (**B**) PavDAM1 or PavDAM5 combined with PavSOC1 in vivo with a bimolecular fluorescence complementation assay in *Nicotiana benthamian*a leaves. Bars, 20 μm.

To further detect the function of sweet cherry PavDAMs in floral transition besides the dormancy, we performed Y2H and BiFC assays to explore the relationship between PavDAMs and PavSOC1. The Y2H assays showed that pGBK-PavDAM1 + pGAD-PavSOC1 and pGBK-PavDAM5 + pGAD-PavSOC1 co-transformed into Y2H cell were able to grow on SD/-Leu/-Trp , SD/-Leu/-Trp/-His/-Ade, and SD/-Leu/-Trp/-His/-Ade with X-α-gal plates (Figure 8A). It proved that PavDAM1 and PavDAM5 interacted with PavSOC1 at the protein level. However, PavDAM4 protein did not interact with PavSOC1 protein (Supplementary Figure S1F). Then the BiFC assay was conducted to verify the interaction of PavDAM1/5 and PavSOC1 in plant cells. PavDAM1- pXY106 + PavSOC1-pXY104 and PavDAM5-pXY106 + PavSOC1-pXY104 were cotransformed into *Nicotiana benthamiana* leaf epidermal cells and yellow YFP fluorescent signals were observed by scanning with confocal laser scanning microscope (Figure 8B).

## 3. Discussion

The sweet cherry DAM gene family are MIKC^c^-type MADS box genes along with those in the peach, leafy spurge, apple, and Japanese apricot [8,10,16,27]. The amino acid sequences of six sweet cherry DAM proteins were highly similar to each other, and there was an extremely high similarity in each pair of DAM proteins between the two cultivars (Figure 2A). The results showed that there were no distinct differences in the amino acid sequences between the low- and high-chill cultivars. Phylogenetic analysis suggested sweet cherry *DAMs* were likely orthologues of *PpDAMs* and *PpsDAMs.* Therefore, we named these genes as *PavDAM1*, *PavDAM2*, *PavDAM3*, *PavDAM4*, *PavDAM5*, and *PavDAM6*. PavDAMs were most closely related to SVP/AGL24, separating from *Arabidopsis* SVP/AGL24 subclade (Figure 2B) [10,28,29].

Previous studies have shown that *DAMs* play an important role in the endodormancy of the bud formation in other species [8,10,16,27]. The distinct seasonal expression patterns of *PavDAMs* genes indicated that, although the *PavDAMs* played the conserved roles in the bud growth, they had the divergent functions in sweet cherry cultivars with different chilling requirement (Figure 3). In both low- and high-chill cultivars, three genes of *PavDAM2*, *PavDAM3* and *PavDAM6* steadily decreased throughout the winter and showed similar patterns, indicating that the three genes played the redundant roles in the dormancy cycles. Compared with the expression profiles of *PavDAM2, PavDAM3* and *PavDAM6*, the transcript levels of *PavDAM1*, *PavDAM4* and *PavDAM5* were higher in the floral buds, suggesting that *PavDAM1/4/5* play primary roles in floral buds dormancy (Figure 3B). The transcript of *PavDAM1* maintained higher level and longer duration in the high-chill cultivar ‘Hongdeng’ in winter and spring, meanwhile, extremely low expression level could be measured in the low-chill cultivar ‘Royal Lee’. Because of a longer duration of higher transcript level of *PavDAM1* in winter in ‘Hongdeng’, they might be responsible for high chilling requirement and the delayed budbreak in spring.

The transcript levels of *PavDAM5* were high and similar in both ‘Royal Lee’ and ‘Hongdeng’, and up-regulated in early winter, subsequently down-regulated in the middle winter (Figure 3B). The elevated transcript levels of *PavDAM5* indicated an important role during the dormancy period, consistent with the peach and Japanese apricot, but not the *PavDAM6* [14,16]. In Japanese apricot, *DAM6* had been confirmed to have the function regulating terminal bud set and growth inhibition in transgenic poplar with high transcript level in buds [16]. However, the low transcript level of *PavDAM6* in both cultivars indicated that it play the redundant roles in the dormancy of sweet cherries. *DAM5* were up-regulated by cold exposure at the endo-dormancy stage through a CBF-binding site (C-repeat/DRE) in the *DAM* promoters [11,14,15]. Ectopic expression of a cold response factor *PpCBF1* in apple induced the growth inhibition and delayed the budbreak [30]. Along with the chilling accumulation, ABA levels increased, which confirmed a relationship with bud dormancy maintenance [31,32]. While the buds chilling accumulation was enough, endogenous ABA levels were down-regulated and GA_3_ level in flower buds were up-regulated during the dormancy release [33,34,35]. Our previous studies showed that hydrogen cyanamide improved GAs:ABA ratio, resulting in the endodormancy release and blooming in sweet cherry [36], and hydrogen cyanamide could down-regulate the expression levels of *DAMs* in peach [15]. The above evidences indicated that ABA and GA were also associated with the expression changes of *PaDAMs.*

Although previous studies have proved that *DAM* genes are associated with endodormancy induction and bud formation in many species, functional and expression analyses of *DAM* genes in sweet cherry showed that they might have a role in flower development. Abnormal flowers observed upon overexpression of *PavDAM1/5* genes in *Arabidopsis* indicated an impact on floral organ identity. 

The *DAM* genes belong to the clade of MIKC^C^ MADS-box gene *SVP* in the peach, and the members of MADS-box gene are expanding in perennials [37]. SVP not only regulates the flowering time as a repressor, but also contributes to the floral transition in *Arabidopsis* [38]. For various perennial species, including kiwifruit, *AcSVP1* and *AcSVP4* delayed budbreak and flowering time in the high-chill cultivar, but not in the low-chill cultivar [39]. Moreover, the overexpression of *SVP-like* genes in plants, including kiwifruit, resulted in abnormal floral phenotypes, such as longer pedicels, leaf-like sepals and deformed siliques [40,41,42,43]. In addition, sterile flowers, misshapen and smaller fruit, and abortive seeds were also observed in the transgenic plants [29,44]. Similarly, the overexpression of *PavDAM1/5* genes in *Arabidopsis* showed abnormal flower phenotypes as well as the *SVP* (Figure 5A–J). The high expression of *PavDAMs* (e.g., *PavDAM1*) in early spring might be responsible for the occurrence of abnormal floral organs in the high-chill sweet cherry cv. ‘Hongdeng’ in warm winter region [6].

To further explore the molecular mechanism of *PavDAMs* regulating the floral development, the relative expression of some genes, including *AP1*, *FUL*, *SEP*, *LYF,* and *SOC1* were investigated. We found that a higher transcript level of *AtSOC1* occurred in the transgenic *Arabidopsis* with overexpression of *PavDAMs* than wild type plants (Figure 6A). *SOC1* regulate the floral transition as an integrator in *Arabidopsis* [22], but in recent studies, it has been affirmed *SOC1-like* genes were associated with chilling requirements and duration of dormancy [45,46,47]. Furthermore, DAM6 has been shown to interact with SOC1 in apricot [21], similar to the interactions between PavDAM1/5 and PavSOC1 (Figure 8), suggesting their possible participation in duration of dormancy, bud formation, and floral organ identity in sweet cherries.

## 4. Materials and Methods

### 4.1. Plant Material 

The sweet cherries ‘Royal Lee’ and ‘Hongdeng’ were selected for the current experiment, which were grafted on Chinese cherry (*P. pseudocerasus* Lindl. Daqingye) rootstock. ‘Royal Lee’ is a low chilling cultivar from the breeding program of low-chill sweet cherries in California, USA, and ‘Hongdeng’ is a high chilling cultivar from China. Both cultivars were grown in the experimental farm at Shanghai Jiao Tong University in Shanghai (31.25°N, 121.48°E), trained to a spindle system, planted at 5 x 6 m spacing and underwent standard orchard management practice. The ambient temperature was recorded by HOBO UX100-003 (HOBO, USA).

Floral buds of sweet cherries were collected on 15 October, 15 November, 15, 30 December in 2017, 15 January, 5, 25 February, 5 March in 2018. All materials were collected for three biological replicates. These buds were frozen in liquid nitrogen and stored at −80 °C before RNA extraction. 

### 4.2. Evaluation of Dormancy Status and Chilling Requirement for Bud Break 

The dormancy status of floral buds at each sampling date was estimated by the following approach. We collected ten 1-year-old shoots from the experimental farm, approximately 50 cm long, and 10–12 floral buds for measuring the percentage of budbreak. The shoots were placed in water in 1 L beakers in a phytotron and kept under a day/night temperature of 25 ± 1/18 ± 1 °C, with a 12-h photoperiod of white light (320 μmol photons m^–2^ s^–1^) and 75% humidity. We changed the water in beaker and cut the basal ends of the shoots every 2–3 d. After 21 d, the dormancy status was evaluated by determining the percentage of budbreak. Floral buds of shoots with budbreak percentages of less than 50% were considered to have remained in the stage of endodormancy [1]. To measure the chilling hours of the two cultivars, the shoots collected on 9 November were taken out from the 4 °C storage house every 200 h, then did as the method above.

### 4.3. Characterization of Sweet Cherry DAM Sequences by Gene Cloning and Phylogenetic Analysis

According to the manufacturer’s instructions, total RNA was extracted using an RNAprep pure Plant Kit (TianGen, China). To isolate the full-length cDNA s of the six *PavDAM* genes, 1 μg of total RNA was converted into cDNA using PrimeScript^TM^ II 1st Strand cDNA Synthesis Kit (TaKaRa Biotechnology, Dalian, China) and was subsequently diluted five times with sterile water. Primers were designed using Primer 5 software according to *DAM* homologs of peach and Chinese cherry [8,19], and sweet cherry reference genome (http://cherry.kazusa.or.jp/). The sequences are listed in Table S1. Then, a PCR amplification was performed with first-strand cDNAs. The PCR-products were cloned into the *pEASY*^®^-Blunt Cloning Vector (TransGen Biotech, Beijing, China), and then sequenced.

Phylogenetic and molecular evolutionary analyses were conducted using MEGA version 5 [48]. To generate a phylogenetic tree, the complete sequences of the other species were obtained from the GenBank DNA database (http://www.ncbi.nlm.nih.gov/genbank/). The Neighbor-Joining method in MEGA was used to construct different trees. The reliability of the obtained trees was tested using bootstrapping with 1000 replicates.

### 4.4. Real-Time Quantitative RT-PCR Analysis

RT-qPCR were performed on a Bio-Rad System (Bio-Rad, CA, USA). The procedure was conducted as follows: 95 °C for 30 s, amplification for 40 cycles (95 °C for 5 s, 60 °C for 30 s). Gene-specific primers for qRT-PCR (Table S1) were designed using Primer 5 software to amplify products between 150–300 bp in size. *PavActin* was used as a reference gene for RT-qPCR analyses. To determine the relative fold differences for each gene in each experiment, the Ct value of the genes was normalized to the Ct value for the reference gene, and the relative expression was calculated relative to a calibrator using the formula 2^−ΔΔCT^ [49]. All the values shown are the mean ± SE.

### 4.5. Subcellular Localization Assessment 

Six *PavDAM* cDNAs were cloned into PHB vectors containing a cauliflower mosaic virus (CaMV) 35S promoter, a translation enhancer and a GFP fluorescent protein tag, respectively. PHB Constructs were transformed into *A. tumefaciens* GV3101 strains and subsequently cultured to an OD600 of approximately 1.0. Leaves of 3 to 5-week-old *Nicotiana benthamiana* plants were infiltrated with the suspension liquid of *A. tumefaciens* GV3101 strains containing the PHB constructs. Localization of fluorescent proteins was monitored 3–7 days after infiltration, the period when GFP fluorescence was optimal, by using a confocal laser scanning microscope (Zeiss LSM510/ConfoCor2). PHB-GFP empty vectors were used as the controls.

### 4.6. Generation of Transgenic Arabidopsis 

The wild-type *Arabidopsis* (Col-0) was used for transformation. Overexpression of *PavDAM1/4/5* and *PavSOC1* were carried out using PHB-based constructs. *Agrobacterium tumefaciens*-mediated plant transformation was performed by the floral dip method [50]. Plants were grown in growth chamber at 21 °C for *Arabidopsis* under a long day (LD) condition (16/8 h, light/dark).

### 4.7. Bimolecular Fluorescence Complementation (BiFC) Assay 

We cloned the 1–516 bp CDS of *PavDAM1*, full length CDS of *PavDAM5* and *PavSOC1* into the vector pXY104 and pXY106 to construct PavDAM1- pXY106, PavDAM5-pXY106 and PavSOC1-pXY104 for BiFC assay. Constructed vectors were transformed into *Agrobacterium tumefaciens* strain GV3101 and subsequently cultured to an OD600 of approximately 0.8–1.0. The mixed suspension liquid with pairs were co-transformed into five-week-old leaves of *Nicotiana benthamiana* after 2 to 5 h. Yellow fluorescent protein (YFP) signals were detected after 48–72 h by a laser scanning confocal microscope (Zeiss LSM510/ConfoCor2). Both pXY104 and pXY106 empty vectors were used as the controls.

### 4.8. Yeast Two-Hybrid (Y2H) Analysis

The 1-516bp CDS of *PavDAM1*, and full length CDS of *PavDAM5* and *PavSOC1* were recombined into the vector pGBKT7 and pGADT7 to create pGBK-PavDAM1, pGBK-PavDAM5 and pGAD-PavSOC1. The two constructs pairs, pGBK-PavDAM1 and pGAD-PavSOC1, pGBK-PavDAM5 and pGAD-PavSOC1, were co-transformed into yeast strain Y2HGold (Clontech). The pGBK-PavDAM1/5 and pGADT7, pGBKT7 and pGAD-PavSOC1 were co-transformed as the negative controls. The transformants were cultured on SD/-Leu/-Trp plates and verified on SD/-Ade/-Leu/-His/-Trp plates (Clontech). The interactions were tested with X-α-gal on SD/-Ade/-Leu/-His/-Trp plates.

## 5. Conclusions

In summary, we cloned the *PavDAM1-6* from sweet cherries, and six *DAM* genes with homology to peach *DAM*, have been identified and characterized from low-chill cultivar ‘Royal Lee’ and high-chill cultivar ‘Hongdeng’. Phylogenetic analysis indicate that these genes are similar to *DAM* in peach, apple and pear. Subcellular localization analysis showed that all genes were localized in the nucleus and cytomembrane. Furthermore, the expression patterns of the *PavDAMs* in the low-chill cultivar ‘Royal Lee’ were different with that in the high-chill cultivar ‘Hongdeng’. ‘Royal Lee’ exhibits lower transcriptional level of *PavDAM1* compared with ‘Hongdeng’, especially at the stage of chilling accumulation, and transcriptional levels of *PavDAM4/5* were high in both cultivars during the endodormancy. In addition, ectopic expression of *PavDAM1* and *PavDAM5* in *Arabidopsis* resulted in plants with abnormal flower and seed development, especially the *PavDAM5*. Higher transcriptional levels of *AtSOC1* were observed in transgenic *PavDAM1/5* lines, and ectopic expression of *PavSOC1* had the similar floral phenotype. Finally, protein interaction analysis demonstrated that PavDAM1/5 could interact with PavSOC1 in vivo and in vitro. Our preliminary results improve our understanding of the mechanism of *PavDAM*-mediated regulation of bud dormancy and flower development in sweet cherry.

## Supplementary Materials

Supplementary materials can be found at https://www.mdpi.com/1422-0067/21/3/921/s1. Constitutive expression of *PavDAM4* does not affect the flower development in *Arabidopsis*. (A,B) Normal flower development of wild-type *Arabidopsis*. (C,D) Normal flower development in *PavDAM4oe Arabidopsis* plants. (E) Relative expression of *PavDAM4* in transgenic and control plants. (** *p* ≤ 0.01; Student’s *t*-test). (F) Yeast two-hybrid assay. pGBK-PaDAM4 did not interact with pGAD-PaSOC1 conferred Y2HGold cell growth on SD/-Leu/-Trp/-His/-Ade plates; Table S1. List of primer sequences used in this study; Table S2. Proteins used for constructing phylogenetic tree and their accession numbers.

## Abbreviations

DAM	Dormancy-associated mads-box
SOC1	Suppressor of overexpression of co1
FT	Flowering locus T
CBF	Cold response genes C-repeat binding factors
AGL24	Agamous-like 24
SVP	Short vegetative phase
