# Supplementary material for: Dormancy-Associated MADS-Box (DAM) Genes Influence Chilling Requirement of Sweet Cherries and Co-Regulate Flower Development with SOC1 Gene
Source: Int J Mol Sci. 2020 Jan 30;21(3):921. doi: 10.3390/ijms21030921 (PMC7037435; doi:10.3390/ijms21030921)
Supplement: Supplementary file 1 [file ijms-21-00921-s001.zip › ijms-693332 suppl for final/Supplementary Tables.pdf]

Supplementary Table 1. List of primer sequences used in this study.

Gene	Forward Primer (5′-3′)	Reverse Primer (5′-3′)
**For gene cloning**		
PavDAM1	ACCAGCAGCGGCAGTCAAC	CAATCACAACCCTCCACTT
PavDAM2	CATTTTAATCCCTTCCTTCG	GCTGGCATGTAATAGATAGGT
PavDAM3	GGGATGGTGAAGATGATGAG	CGTTATCCTGTGCTGTGAC
PavDAM4	AACGAAGGGGGATGGTGAAAATGAAG	CTTAACCAAGGAAATTATGGACGCC
PavDAM5	AACCCACCAACGAAGATGGTGAGG	CTTTCTCCACTTCTTTAACGCCCCA
PavDAM6	GACAAAGGGAAACGGTGAA	TCCACTTCTTAACTAGGAAACTAGG
PavSOC1	CGCTTCCTTCTGTTAAAAATGGTGAGAGG	TCTGAGGAGAAGCTAGCGCTTCACTCTTC
**For real-time qPCR**		
PavACTIN	CGGTATTGCAGACGGATGAGC	GGTACTGAGGGATGCAAGGATGG
PavDAM1	AGGGGACGATGAAAATGATGAGG	GATAACTGCCACCTCAGATTCACA
PavDAM2	GTTAGAACAACTGGTGGACGCAAG	AGCTGCTGATGGTTGCATTTGTGG
PavDAM3	AGCCAACAACCAGTTAAGGCAGAC	GTCATCTCCAAGAGAGTGAGCACT
PavDAM4	AGCCAACAACCAGTTAAGGCAGAC	CAAGAGAGAGACTGAGAGCACTGTT
PavDAM5	AATTGAATGATCAAGAGATTGAA	CAGCTCTTCCTTAGTTTCCATG
PavDAM6	GAGTGAGATCATGTCACTGGAGAA	AGCTGGTAGAGGTGGCCATTGTG
PavSOC1	TCTCTGTGATGCTGAGGTTGC	TTTTGGTCAGTGGAAACGGAT
AtACTIN	GGTAACATTGTGCTCAGTGGTGG	AACGACCTTAATCTTCATGCTGC
AtSOC1	AGCTGCAGAAAACGAGAAGCTCTCTG	GGGCTACTCTCTTCATCACCTCTTCC
**For subcellular localization**		
PavDAM1	ACCAGTCTCTCTCTCAAGCTTATGAAAATG ATGAGGGAGAAG	GCCCTTGCTCACCATGGATCCTAAAAGCCCC AGTTTGAGAGA
PavDAM2	ACCAGTCTCTCTCTCAAGCTTATGGTGAAG ATGATGAGGAAG	GCCCTTGCTCACCATGGATCCGGGAAGCCCC AGTTTGAGAGC
PavDAM3	ACCAGTCTCTCTCTCAAGCTTATGGTGAAG ATGATGAGGAAG	GCCCTTGCTCACCATGGATCCGGGAAGCCCC AGTTTGAGAGA
PavDAM4	ACCAGTCTCTCTCTCAAGCTTATGGTGAAA ATGAAGAGGGAG	GCCCTTGCTCACCATGGATCCTGGACGCCCTA ATTTGAGAGA
PavDAM5	ACCAGTCTCTCTCTCAAGCTTATGATGAGG AATAAGATCAAG	GCCCTTGCTCACCATGGATCCACGCCCCAGTT TGAGGGATAA
PavDAM6	ACCAGTCTCTCTCTCAAGCTTATGATGAGG GAAAAGATCAAA	GCCCTTGCTCACCATGGATCCGGGAAGCCCC AATTTGAGAGA
**For transgenic plants**		
PavDAM1	ACCAGTCTCTCTCTCAAGCTTATGAAAATG ATGAGGGAGAAG	GCAGCTCGAGGATCCAAGCTTTTATAAAAGC CCCAGTTTGAG
PavDAM4	ACCAGTCTCTCTCTCAAGCTTATGGTGAAA ATGAAGAGGGAG	GCAGCTCGAGGATCCAAGCTTTTATGGACGC CCTAATTTGAG
PavDAM5	ACCAGTCTCTCTCTCAAGCTTATGATGAGG AATAAGATCAAG	GCAGCTCGAGGATCCAAGCTTTTAACGCCCC AGTTTGAGGGA
PavSOC1	ACCAGTCTCTCTCTCAAGCTTATGGTGAGA GGAAAAACCCAG	GCAGCTCGAGGATCCAAGCTTCTAGCGCTTC ACTCTTCTTTC
**For yeast two-hybrid assay**		
PavDAM1	TCAGAGGAGGACCTGCATATGATGAAAAT GATGAGGGAGAAG	TCGACGGATCCCCGGGAATTCCATCCTCTGCC TTAGCTGTTT
PavDAM4	TCAGAGGAGGACCTGCATATGATGGTGAA AATGAAGAGGGAG	TCGACGGATCCCCGGGAATTCTTATGGACGC CCTAATTTGAG
PavDAM5	TCAGAGGAGGACCTGCATATGATGATGAG GAATAAGATCAAG	TCGACGGATCCCCGGGAATTCTTAACGCCCC AGTTTGAGGGA
PavSOC1	GTACCAGATTACGCTCATATGATGGTGAGA GGAAAAACCCAG	ATGCCCACCCGGGTGGAATTCCTAGCGCTTC ACTCTTCTTTC
**For bimolecular fluorescence complementation**		
PavDAM1	GACGCCGGCGGATCCTCTAGAATGAAAAT GATGAGGGAGAAG	GCTCTGCAGGTCGACTCTAGACATCCTCTGCC TTAGCTGTTT
PavDAM5	GACGCCGGCGGATCCTCTAGAATGATGAG GAATAAGATCAAG	GCTCTGCAGGTCGACTCTAGATTAACGCCCC AGTTTGAGGGA
PavSOC1	GGTACCCGGGGATCCTCTAGAATGGTGAG AGGAAAAACCCAG	GCCACCGCCGTCGACTCTAGAGCGCTTCACT CTTCTTTCTGG

Supplementary Table 2. Proteins used for constructing phylogenetic tree and their accession numbers

**Protein Name**	**NCBI Accession Number**
Peach *(Prunus persica)*	
PpDAM1	DQ863253
PpDAM2	DQ863255
PpDAM3	DQ863256
PpDAM4	DQ863250
PpDAM5	DQ863251
PpDAM6	DQ863252
PpSVP	LOC18790353
Plum(*Prunus mume*)	
PmAGL24-like	LOC103319498
Chinese Cherry(*Prunus pseudocerasus*)	
PpsDAM3	KM243364
PpsDAM4	KM243365
PpsDAM5	KM243366
PpsDAM6	KM243367
Sweet Cherry(*Prunus avium*)	
PavMADS1	EU196362
Pear (*Pyrus pyrifolia* or *Pyrus x bretschneideri)*)	
PpyMADS13-1	AB504716
PpyMADS13-2	AB504717
PpyMADS13-3	AB774474
PpyDAM3-like	KP164028
PpyMADS1	KC261355
PpyMADS2	KC261356
PpyMADS13-3hh	KU844854
PbAGL24-like	LOC103964948
Apple (*Malus x domestica*)	
MdDAM1	KT582786
MdDAM2	KT582787
MdDAM4	KT582789
MdSVP-like	LOC103416871
Jujube(*Ziziphus jujuba*)	
ZjSVP-like	LOC107421277
Poplar(*Populus tomentosar*)	
PtMADS	AY501392
Walnut(*Juglans regia* or *Carya cathayensis*)	
JrSVP-like	LOC109013707

CcSVP-like	KJ546348
CcAGL24	KF918308
Arabidopsis thaliana	
AtSVP	AF211171
AtAGL24	NM118587
