# Supplementary figures and images for: Dormancy-Associated MADS-Box (DAM) Genes Influence Chilling Requirement of Sweet Cherries and Co-Regulate Flower Development with SOC1 Gene
Source: Int J Mol Sci. 2020 Jan 30;21(3):921. doi: 10.3390/ijms21030921 (PMC7037435; doi:10.3390/ijms21030921)

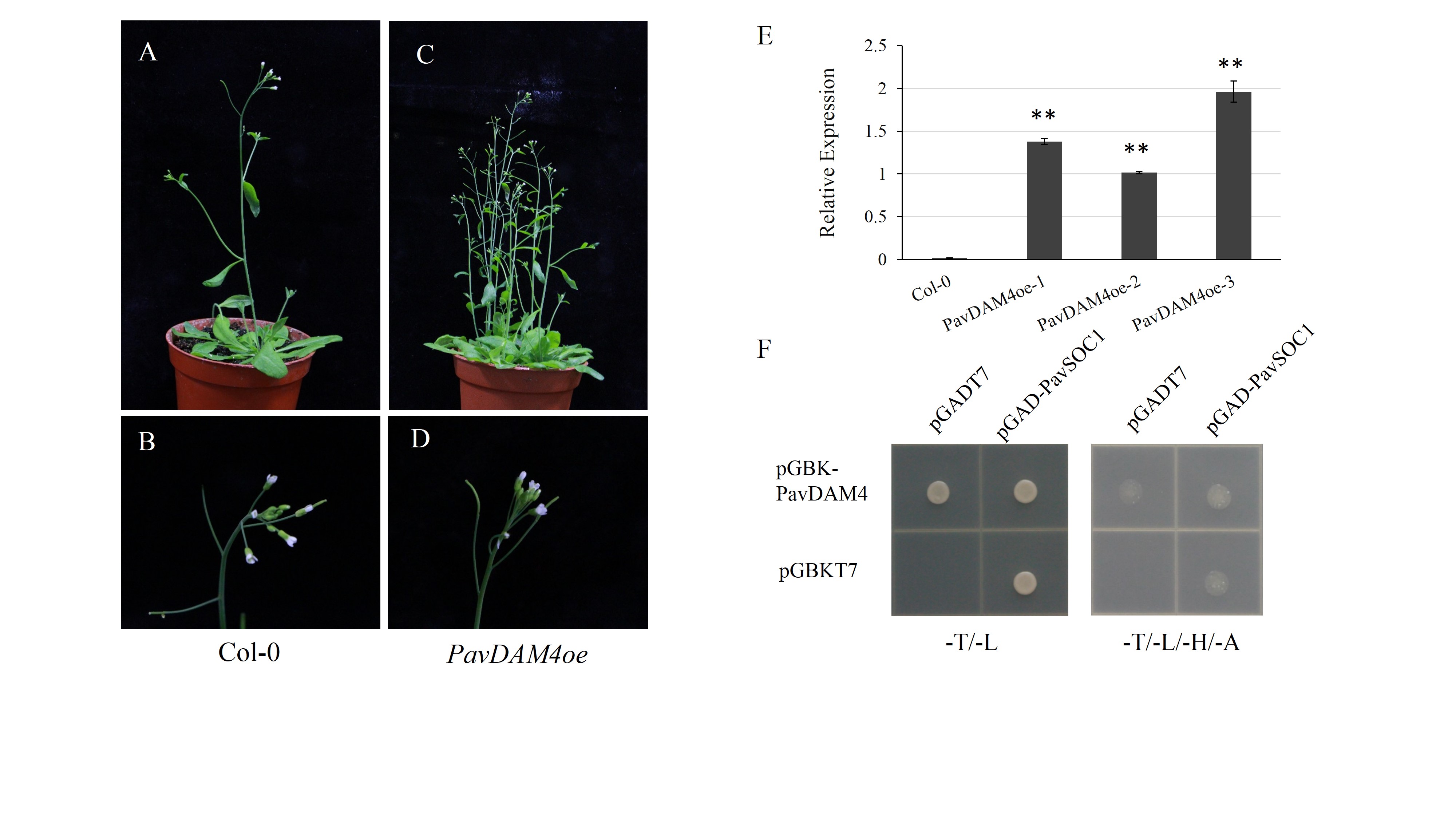

Supplement: Supplementary file 1 [file ijms-21-00921-s001.zip › ijms-693332 suppl for final/Supplementary Fig. 1.jpg]
